# Geometry-Aware Human Noise Removal from TLS Point Clouds via 2D Segmentation Projection

**DOI:** 10.3390/s26041237

**Published:** 2026-02-13

**Authors:** Fuga Komura, Daisuke Yoshida, Ryosei Ueda

**Affiliations:** 1 Graduate School of Informatics, Osaka Metropolitan University, Osaka 558-8585, Japan; 2 College of Sustainable System Sciences, Osaka Metropolitan University, Osaka 599-8531, Japan

**Keywords:** 3D point cloud, noise removal, image recognition, deep learning, principal component analysis, TLS, DBSCAN

## Abstract

Large-scale terrestrial laser scanning (TLS) point clouds are increasingly used for applications such as digital twins and cultural heritage documentation; however, removing unwanted human points captured during acquisition remains a largely manual and time-consuming process. This study proposes a geometry-aware framework for automatically removing human noise from TLS point clouds by projecting 2D instance segmentation masks (obtained using You Only Look Once (YOLO) v8 with an instance segmentation head) into 3D space and validating candidates through multi-stage geometric filtering. To suppress false positives induced by reprojection misalignment and planar background structures (e.g., walls and ground), we introduce projection-followed geometric validation (or “geometric gating”) using Density-Based Spatial Clustering of Applications with Noise (DBSCAN) and principal component analysis (PCA)-based planarity analysis, followed by cluster-level plausibility checks. Experiments were conducted on two real-world outdoor TLS datasets—(i) Osaka Metropolitan University Sugimoto Campus (OMU) (82 scenes) and (ii) Jinaimachi historic district in Tondabayashi (JM) (68 scenes). The results demonstrate that the proposed method achieves high noise removal accuracy, obtaining precision/recall/intersection over union (IoU) of 0.9502/0.9014/0.8607 on OMU and 0.8912/0.9028/0.8132 on JM. Additional experiments on mobile mapping system (MMS) data from the Waymo Open Dataset demonstrate stable performance without parameter recalibration. Furthermore, quantitative and qualitative comparisons with representative time-series geometric dynamic object removal methods, including DUFOMap and BeautyMap, show that the proposed approach maintains competitive recall under a human-only ground-truth definition while reducing over-removal of static structures in TLS scenes, particularly when humans are observed in only one or a few scans due to limited revisit frequency. The end-to-end processing time with YOLOv8 was 935.62 s for 82 scenes (11.4 s/scene) on OMU and 571.58 s for 68 scenes (8.4 s/scene) on JM, supporting practical efficiency on high-resolution TLS imagery. Ablation studies further clarify the role of each stage and indicate stable performance under the observed reprojection errors. The annotated human point cloud dataset used in this study has been publicly released to facilitate reproducibility and further research on human noise removal in large-scale TLS scenes.

## 1. Introduction

Recent advances and cost reductions in laser scanning have made the acquisition of 3D point clouds more accessible than ever. In addition to Light Detection and Ranging (LiDAR) now being available on smartphones and tablets, the growing use of terrestrial laser scanners (TLSs) and mobile mapping systems (MMSs) has broadened applications across cultural heritage documentation and digital twins [1]. For cultural assets, automatically classifying large-scale point clouds into constituent elements (e.g., buildings, vegetation, and humans) remains a significant challenge [2]. At the same time, removing non-target points unintentionally captured during surveying (hereafter referred to as “noise”, e.g., pedestrians or vehicles) is a critical preprocessing step that directly affects the accuracy and efficiency of downstream tasks such as modeling, geometric analysis, and temporal comparison [3]. In current practice, noise removal is still largely manual; at city or building scales with billions of points, the workload becomes prohibitive.

Motivated by these gaps, this study proposes a TLS-oriented human noise removal pipeline that projects 2D instance segmentation masks obtained using You Only Look Once (YOLO) v8, implemented with the Ultralytics framework (version 8.3.63) and pretrained on the Common Objects in Context (COCO) 2017 dataset [4], into point-cloud space, and then suppresses false positives using geometry-aware filtering based on principal component analysis (PCA)-derived planarity, local height range, and Density-Based Spatial Clustering of Applications with Noise (DBSCAN)-based cluster properties [5]. Our main contributions are as follows: (1) we used an annotation-efficient strategy that avoids training a 3D network by leveraging widely available pretrained 2D models; (2) a post-projection geometric validation step that reduces false positives induced by 2D–3D misalignment and background structures; and (3) a practical workflow that improves large-scale TLS processing by reducing manual effort. The novelty of this research lies in the projection-followed, geometry-aware validation (gating) that systematically suppresses false positives induced by 2D–3D misalignment without training any 3D network. In addition, we provide the manually annotated human point cloud dataset used in our experiments to facilitate further research on noise removal in TLS point clouds.

## 2. Related Work

Deep learning for point-cloud processing has rapidly progressed, spanning methods that operate directly on points, voxel-based representations, and projections to 2D images [6]. From a methodological perspective, these studies can be categorized into commonly used methods, such as learning-based, detection-based, and clustering- or geometry-driven approaches. Related studies can be organized into three groups. In addition, recent work has addressed dynamic point removal for static map construction from repeated LiDAR scans.

(i) Point-cloud-native learning-based methods. Since PointNet [7], numerous architectures have been proposed for the semantic segmentation and understanding of large-scale point clouds, including RandLA-Net [8], KPConv [9], Point Transformer [10], Stratified Transformer [11], MinkowskiNet [12], and PointNeXt [13]. While these models achieve strong performance, they generally require large amounts of 3D annotations for supervised training, which can be expensive and time-consuming to obtain in practical surveying workflows [6,8]. In addition, surveys on point-cloud denoising and processing provide broad overviews of such learning-based pipelines and their challenges in real-world scenes [3,6]. More recently, newer point-based backbones have continued to improve the efficiency–accuracy trade-off on large-scale scenes; for example, Point Transformer V3 (PTv3) provides a streamlined and scalable architecture for point cloud understanding [14]. Complementary to learning-based backbones, practical large-scale point-cloud workflows often employ lightweight geometric preprocessing to reduce the search space and improve computational efficiency. Such grid-based preprocessing is widely used in tasks such as ground-point extraction.
(ii) Image-based detection/segmentation models. High-accuracy detectors and instance segmentation frameworks such as Mask Region-Based Convolutional Neural Network (Mask R-CNN) [15] and one-stage detectors, such as YOLO [16], have been widely adopted in 2D vision. Recent implementations of Ultralytics (e.g., YOLOv8) also provide instance segmentation variants suitable for efficient mask extraction [17]. Large-scale datasets such as COCO [4] have made pretrained models widely available. For semantic segmentation, models such as DeepLab [18] are commonly used, while transformer-based detectors (e.g., DETR) [19] further broaden the design space for robust 2D recognition. In addition, modern mask prediction transformers and foundation segmentation models (e.g., Mask2Former and Segment Anything) have advanced general-purpose mask extraction, offering strong 2D priors that can be integrated into 2D–3D workflows when appropriate [20,21].
(iii) Examples of 2D–3D fusion and projection-based coupling. Several frameworks propagate 2D recognition results into 3D space to gain a broader understanding. Frustum PointNets [22] and RoarNet [23], for example, generate 3D frustums from 2D detections and apply dedicated 3D networks within them. PointPainting assigns image-segmentation cues to points to improve 3D object detection [24]. These methods share the general direction of transferring 2D recognition into 3D, but they typically do not develop post-projection mechanisms that systematically suppress false positives using explicit geometric consistency checks. By contrast, our approach combines pretrained 2D segmentation with geometry-aware filtering, leveraging planarity-related cues [25] and density-based clustering principles [5,26] to reduce false positives caused by projection misalignment and background structures. Recent fusion methods have explored tighter cross-modal coupling. This can be performed, for example, via cross-attention between multi-view image features and 3D point features (e.g., 2D–3D Interlaced Transformer) [27] and open-vocabulary co-embedding of points, images, and text to understand flexible 3D scenes (e.g., OpenScene) [28]. Recently, Yue et al. [29] reported a YOLO-based 2D–3D fusion approach to improve multi-class point cloud segmentation.

Recent (2023–2025) studies have explicitly targeted dynamic/transient point removal to build clean static maps from LiDAR scans. Representative approaches include DynaHull [30], which filters dynamic points by exploiting density changes across multiple scans, and DUFOMap [31] and BeautyMap [32], which suppress transient points via scan-to-map consistency and visibility reasoning. While these methods are closely related to our target task in that they aim to remove human-induced artifacts from point clouds, they typically assume sequential or multi-pass observations to accumulate temporal evidence. In contrast, our focus is on single-station TLS scans accompanied by co-registered RGB images, where temporal redundancy is limited. Therefore, we use 2D instance masks primarily to propose candidates and then apply projection-followed, geometry-aware validation to robustly reject false positives under reprojection misalignment and planar background structures. We further include a direct comparison with a recent dynamic point removal baseline (DUFOMap and BeautyMap) in Section 5.2.2.

Most recent “dynamic object removal” methods are designed for sequential LiDAR observations and exploit temporal consistency across multiple frames to build a static map. In contrast, our target setting is single-station static TLS scans, where temporal cues are not available and the image–point correspondence is established per scan station. Consequently, publicly available state-of-the-art baselines that strictly match this static TLS setting are limited. Given this constraint, we selected DUFOMap and BeautyMap as representative recent methods with accessible implementations and well-documented evaluation protocols, and we report their performance and runtime alongside our approach to provide a practical and transparent reference point.

## 3. Proposed Method

### 3.1. Overview

**Figure 1:**
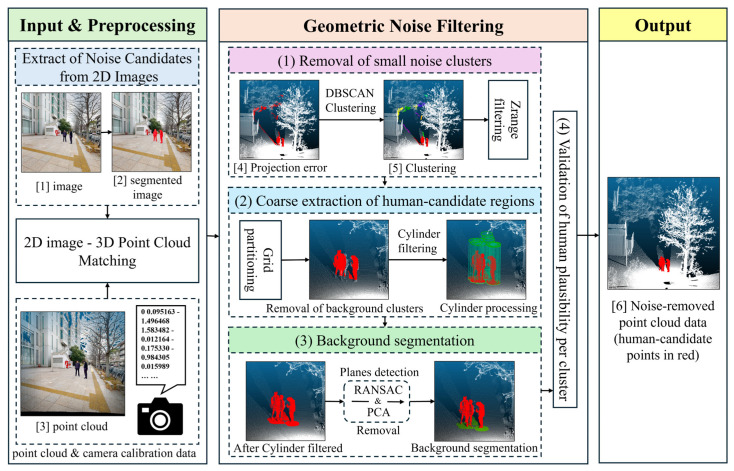
Processing pipeline for automatic human noise removal from terrestrial laser scanning (TLS) point clouds. Detailed visualizations of each step are provided in Figures 2–4 and 7.

We propose a method that combines deep-learning-based image recognition with geometric feature analysis of point clouds. This approach can enhance both the efficiency and accuracy of noise removal in three-dimensional (3D) point-cloud data. Leveraging the fact that many 3D laser scanners acquire images and point clouds for colorization, we used image recognition to identify noise candidates in the 2D images before matching them to the point cloud. Next, we applied geometric processing, such as principal component analysis (PCA) and clustering, to the corresponding candidate points to better discriminate humans from background structures and effectively suppress false positives. An overview of the processing pipeline is shown in Figure 1. In the following sections, each step is described in detail.

### 3.2. Extraction of Noise Candidates from 2D Images (Input and Preprocessing)

**Figure 2:**
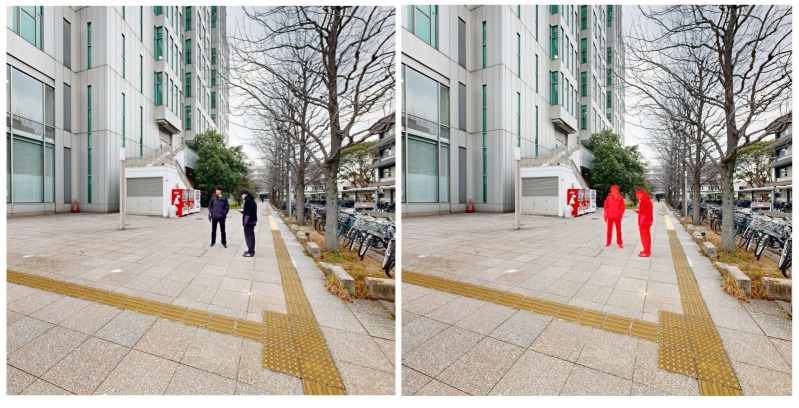
Instance segmentation results using You Only Look Once (YOLO) v8-seg. (**Left**): input image; (**Right**): detected humans shown in red.

In the first stage of our method, we leverage the 2D red–green–blue (RGB) images acquired with the TLS point cloud to identify human-related noise candidates at the pixel level (Figure 2). We apply YOLOv8-seg (Ultralytics), a one-stage object detector equipped with an instance segmentation head, to each image and obtain a person instance mask for every detected individual. The set of pixel coordinates inside these masks is then used in the subsequent 2D–3D matching step (Section 3.3) to retrieve the corresponding 3D points. This restricts the following geometric processing to regions likely to contain humans.

### 3.3. The 2D Image–3D Point Cloud Matching Stage (Input and Preprocessing)

**Figure 3:**
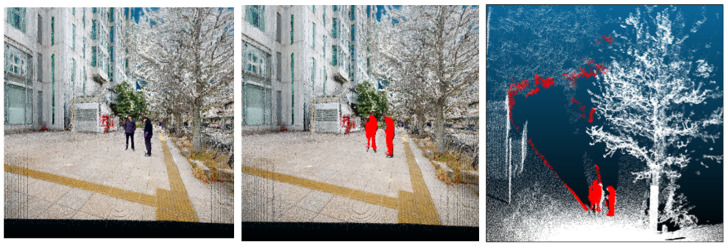
Results of 2D image–3D point cloud matching. (**Left**): Original point cloud. (**Center**): Matched points shown in red. (**Right**): Matched points shown in red from a different angle.

The two-dimensional candidate regions (segmentation masks) identified in Section 3.2 are associated with the three-dimensional point-cloud data (Figure 3). This is achieved via a coordinate transformation using the intrinsic and extrinsic parameters. Specifically, each 3D point in world coordinates is projected onto the camera image plane using Equation (1) to obtain its pixel coordinates.

(1) $$s\left[\begin{array}{c} u\\ v\\ 1 \end{array}\right]=K\cdot R\cdot \left(\left[\begin{array}{c} X\\ Y\\ Z \end{array}\right]-t\right)$$

Here, K denotes the camera intrinsic matrix, R is the rotation matrix, and t is the translation vector; *u* and *v* are the pixel coordinates on the image, and *s* is a scaling factor. We apply this computation to all points in the candidate set and retain only those with projected pixel coordinates that fall inside the person instance masks predicted by YOLOv8-seg (Section 3.2). In this way, we accurately identified the 3D points corresponding to each mask and narrowed down the targets for subsequent geometric processing. The specific acquisition and validation of these parameters in our experimental setup are detailed in Section 4.1.4.

### 3.4. False-Positive Suppression via Geometric Processing

After the 2D–3D matching in Section 3.3, the resulting candidate set often still contains background points due to reprojection/calibration offsets as well as mask over-coverage (Figure 3). To suppress these false positives while maintaining a high recall, we apply the following four-step geometric filtering pipeline: (i) removal of small noise clusters via DBSCAN and Z-extent screening; (ii) coarse region restriction by grid partitioning and a human-sized cylindrical filter; (iii) background segmentation using geometric cues to reject dominant planar structures; and (iv) per-cluster human plausibility validation to remove remaining non-human clusters. The output of this section is the final set of points to be removed as human-related noise.

#### 3.4.1. Removal of Small Noise Clusters

To improve the accuracy and efficiency of subsequent stages, preprocessing steps include first removing clear non-human small noise clusters from the candidate point sets obtained by the matching in Section 3.3. In the image–point cloud matching process, slight errors in camera parameters or calibration inevitably introduce small positional offsets between the 2D masks and the 3D points. Consequently, human masks may slightly overlap with background objects, such as walls, trees, or the ground, causing these background points to be erroneously identified as humans (see the right panel of Figure 3).

**Figure 4:**
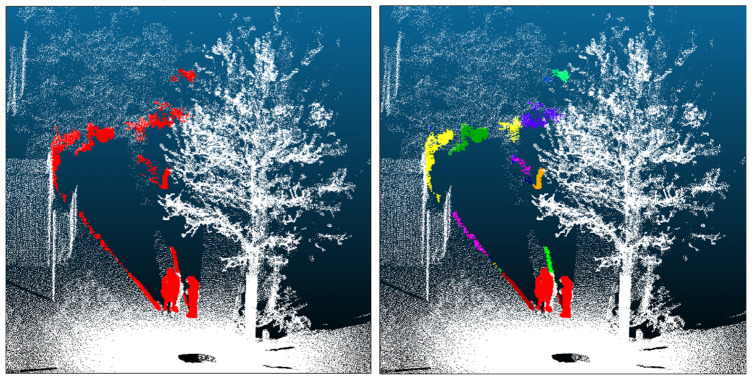
Separation of false positives via clustering. (**Left**): Candidate points before clustering. (**Right**): Clusters after Density-Based Spatial Clustering of Applications with Noise (DBSCAN). Red indicates retained human candidate clusters; non-red colors denote clusters removed by the Z-extent criterion (<0.25 m).

These false-positive points typically appear as small, spatially separated clusters that are distinct from actual human geometry, and they share a common property of having a limited extent along the *Z*-axis. Therefore, before detailed shape analysis, we cluster the candidate points using DBSCAN (Figure 4). We set the neighborhood radius to ε = 0.25 m and the minimum number of points to min_samples (MinPts) = 6; the parameter validation is reported in Section 4.3.1. We then compute the height of each cluster in the Z direction and remove clusters with a Z-extent lower than 0.25 m. This threshold is chosen empirically to exceed typical heights of the background artifacts to be removed (e.g., ground or curbs), while minimizing the risk of mistakenly discarding human-related clusters (e.g., crouching poses or partial body segments). In Figure 4(right), clusters shown in colors other than red are those removed by this criterion.

#### 3.4.2. Coarse Extraction of Human Candidate Regions

**Figure 5:**
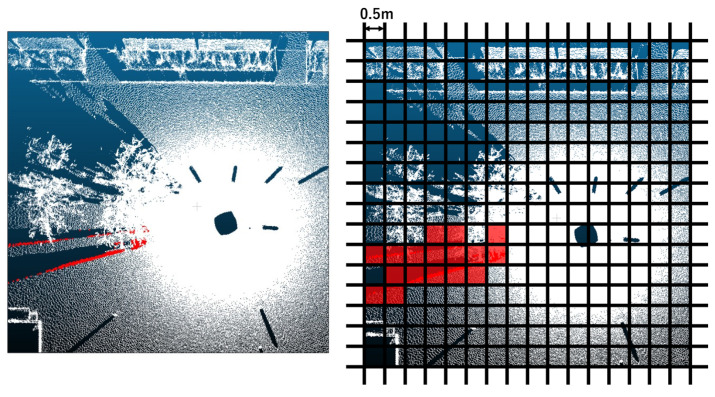
Restricting human candidate regions via grid partitioning. (**Left**): XY-plane view of the input 3D point cloud (red indicates point sets containing human candidates). (**Right**): Grids containing human candidates highlighted in red.

Next, we perform a coarse extraction step to restrict subsequent geometric processing to localized regions likely to contain humans. After the preprocessing step in Section 3.4.1, we partition the XY plane into 0.5 m × 0.5 m grids and keep only the grids that contain any projected human candidate points (Figure 5). This spatial indexing confines later computations within each grid, substantially reducing the computational cost of large-scale scans.

Next, we apply filtering to each grid. For grids identified to contain human candidates, we first evaluate the *Z*-axis height range (Z-range) of all points in the grid. If this height is less than 1.0 m, the grid is determined to contain a ground-level or a very low structure, and all points are classified as non-human. We used 1.0 m as a conservative cutoff to discard ground and other low-lying structures while retaining pedestrian-scale objects; a stricter plausibility check is applied later in Section 3.4.4.

For grids that satisfy this height condition, we further apply a cylindrical filter. We compute the arithmetic means of the X and Y coordinates of the human candidate points in the grid to obtain the centroid ($C_{x}$, $C_{y}$) (Equations (2) and (3)) and define a virtual cylinder centered at this point. The cylinder radius is set to r = 0.35 m based on the parameter validation described in Section 4.3.1, and its height is set to 2.5 m, which covers the expected human height range and provides tolerance for posture variation and minor projection/calibration errors. Only points lying inside this cylinder are retained as “human candidates,” while all points outside the cylinder are removed as non-human.

(2) $$C_{x}=\frac{1}{n}\sum_{i=1}^{n}x_{i}$$

(3) $$C_{y}=\frac{1}{n}\sum_{i=1}^{n}y_{i}$$

#### 3.4.3. Background Segmentation

Even after coarse extraction, the human candidate point sets may still include large background structures such as walls and ground. We therefore perform geometry-based segmentation to suppress false positives [25]. First, to improve wall detection, we build a high-density wall detection set by augmenting not only the current candidate points but also all points within a 1 m radius around them.

We then apply the random sample consensus (RANSAC) algorithm [33] to this high-density set to extract up to 20 dominant planes (iterations limited to 20). However, RANSAC alone can mistakenly fit planes to side-by-side humans or distant humans that appear locally planar. To verify that a detected plane is geometrically well-formed, we compute a planarity score on its inlier points using PCA of local neighborhoods [34]. The plane is accepted only if the calculated planarity score exceeds 0.6; the parameter validation is reported in Section 4.3.2. Planes that pass this test are labeled as walls.

**Figure 6:**
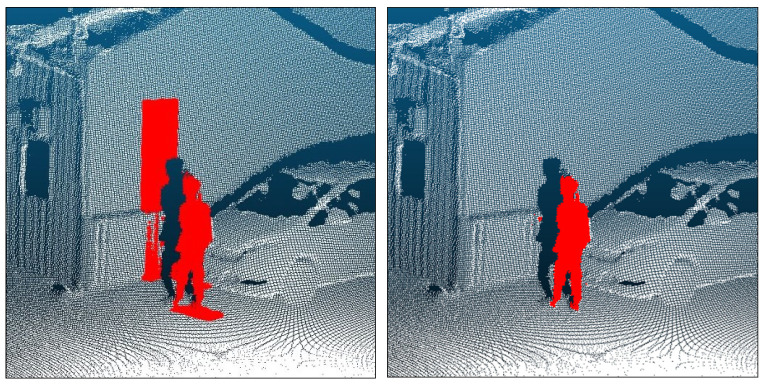
Removal of walls/ground using principal component analysis (PCA). (**Left**): Without PCA—humans are highlighted in red; background structures are highlighted in gray. (**Right**): With PCA—planar background removed; remaining human candidate points are shown in red.

Finally, to remove fine wall corrugations and nearby wall points that may have been missed by fitting, we define the wall core as the overlap between the accepted walls and the original candidate set. We then dilate the wall core by a 0.15 m buffer and remove all candidate points within this radius as instances of wall/background. This procedure enables high-precision removal of background structures based on 3D shape cues that are difficult to reject by image recognition alone (see Figure 6).

#### 3.4.4. Validation of Human Plausibility per Cluster

Even after background structures are removed, the remaining human candidate points may still form a single mass that mixes multiple nearby people with non-human objects (e.g., parts of vehicles or vegetation). In such an undivided state, it is difficult to assess plausible human forms from the overall image. Therefore, we reapply DBSCAN—using the same clustering method and parameter settings as in Section 3.4.1 (ε = 0.25 m, min_samples = 6)—to separate the candidate mass into individual clusters (“instance separation”).

**Figure 7:**
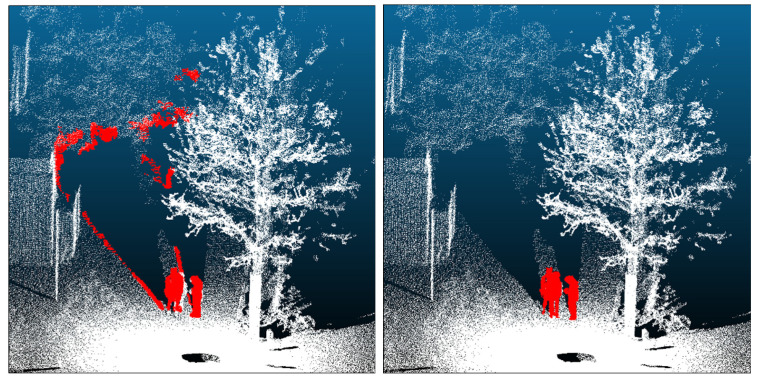
Results of false-positive suppression via geometric processing. (**Left**): Before suppression. Human points and falsely detected background regions are highlighted in red; the background is shown in gray. (**Right**): After suppression. Falsely detected background points are removed (red disappears), and background structures remain in gray.

We then validate each cluster using two plausibility checks to further reduce false positives. (i) Spatial context filtering: We compute the 3D distance from the scanner position to each cluster centroid and discard clusters beyond 30 m, where point clouds become sparse and shape reliability deteriorates; the choice of this threshold is validated in Section 4.3.3. (ii) Geometric consistency filtering: We compute each cluster’s Z-extent and retain only those with height that fall within a realistic human range (1.0–2.2 m in this study). Clusters satisfying both checks are finally confirmed as human noise. Figure 7 illustrates that falsely detected background regions are removed while true human clusters are preserved.

## 4. Experiments

### 4.1. Experimental Setup and Datasets

#### 4.1.1. Data Acquisition and Datasets

The 3D point clouds and images used in this study were captured with a Matterport Pro3 scanner. Matterport Pro3 supports a wide range of areas up to 100 m, with an accuracy of approximately ±20 mm at 10 m, acquiring a high-density point cloud of 1.5 million points per scan under the standard density setting. To evaluate the proposed method under different sensor configurations and environmental conditions, we prepared two TLS datasets and additionally incorporated one MMS dataset.

- Osaka Metropolitan University, Sugimoto Campus (hereafter, OMU)—60 stations and 82 scenes. This campus has substantial daily pedestrian traffic, making it well-suited for evaluating human detection performance (i.e., human-related noise).
- Jinaimachi, Tondabayashi City, Osaka Prefecture, Japan (hereafter, JM)—55 stations and 68 scenes. Designated as an Important Preservation District for Groups of Traditional Buildings, this area contains many planar structures, such as walls; it is, therefore, appropriate for testing the suppression of false positives using practical cultural-heritage scenarios.
- Waymo Open Dataset (Perception Dataset v2.0.1) [35]. We use the validation split of the Waymo Open Dataset as the evaluation dataset, from which 15 scenes were randomly selected. This dataset was captured using a vehicle-mounted MMS and thus differs substantially from the TLS datasets in terms of sensor motion, continuously changing viewpoints, and illumination variations; therefore, it is used here to examine the transferability and limitations of the proposed method beyond static TLS settings.

#### 4.1.2. Ground-Truth Annotation

For quantitative evaluation, we prepared a point-level ground truth for human-related noise. Specifically, human points were manually annotated for each TLS scene by visually inspecting the 3D point cloud (geometry and color information) using the Segment tool and Cloud Layers feature in CloudCompare (version 2.14.alpha). The Matterport Pro3 (Matterport, Inc., Sunnyvale, CA, USA) captures four distinct high-resolution images at each station, which collectively cover a full 360-degree horizontal field of view. This hardware configuration ensures that every point in the 3D point cloud has a corresponding 2D image for reference. Leveraging this multi-directional coverage, we annotated all humans within the entire 360-degree range. To ensure precise spatial correspondence between the 2D detections across the four images and the 3D point cloud geometry, our annotation targeted pedestrians who remained static during the capture process, while individuals in motion were excluded from the ground truth (GT). To maintain objectivity, we established clear criteria for identifying “human” points. In addition to the main body of the pedestrian, we explicitly included personal belongings (e.g., backpacks). These items were included because excluding them while removing only the human body would leave unnatural floating artifacts in the final point cloud, which is counterproductive for practical surveying and engineering workflows.

All non-annotated points in the scene were treated as background for computing the evaluation metrics in Section 4.2 (true positives (TP), false positives (FP), and false negatives (FN)).

To ensure consistency, we labeled the visible portions of humans even when they were partially occluded or truncated, while avoiding the inclusion of surrounding structures (e.g., walls, vegetation, or ground) that may be mixed due to projection or alignment imperfections.

The resulting manual annotations of human points used for the evaluation are publicly available as an open dataset on GitHub (see the Data Availability Statement for the URL). Note that the released resource only includes TLS scenes containing human noise annotations and does not represent the complete set of raw TLS scans acquired during data collection.

#### 4.1.3. Implementation Details (2D Inference and Computing Environment)

We used the publicly available YOLOv8-seg model pretrained on COCO 2017 (Ultralytics repository). All 2D inference experiments were conducted on an NVIDIA GeForce RTX 2080 Ti GPU (NVIDIA Corporation, Santa Clara, CA, USA). To accommodate the high spatial resolution of TLS images under GPU memory constraints, we evaluated multiple inference configurations, including variants that had tiling enabled. These configurations were assessed as part of the comparative experiments described in Section 4.3.4. The final inference setting adopted in the proposed method is discussed in detail in that section.

#### 4.1.4. Evaluation of Reprojection Accuracy

Because the RGB images are captured by the scanner’s built-in cameras at each static scan station, the images and point cloud are naturally time-aligned in our setup; thus, the primary source of mismatch is reprojection (calibration) error rather than temporal desynchronization. Because our 2D–3D association relies on the estimated intrinsic and extrinsic parameters, we evaluated their practical accuracy via reprojection error analysis. The intrinsic parameters were estimated for the camera units integrated into the Matterport Pro3 scanner using the “Camera Calibrator” application in MATLAB R2024b. During data acquisition, the scanner simultaneously captures four high-resolution color images (4096 × 4096 pixels) at each scan location, forming a skybox that covers four horizontal directions (front, back, left, and right) and two vertical directions (up and down). In this study, only horizontal images were used because human subjects predominantly appear in these views. For calibration, an A1-sized checkerboard with a known grid size of 35 mm was mounted on a rigid board to avoid bending effects, and 16 images from different viewpoints were used to estimate the focal lengths $\left(f_{x},f_{y}\right)$, principal point coordinates $\left(c_{x},c_{y}\right)$, and lens distortion coefficients. The extrinsic parameters for each scan were extracted from the E57-format files generated after acquisition, which provide the scanner position (X,Y,Z) and orientation as a quaternion $\left(q_{w},q_{x},q_{y},q_{z}\right)$. Based on these values, the rotation matrix R and translation vector t for each image were constructed.

**Figure 8:**
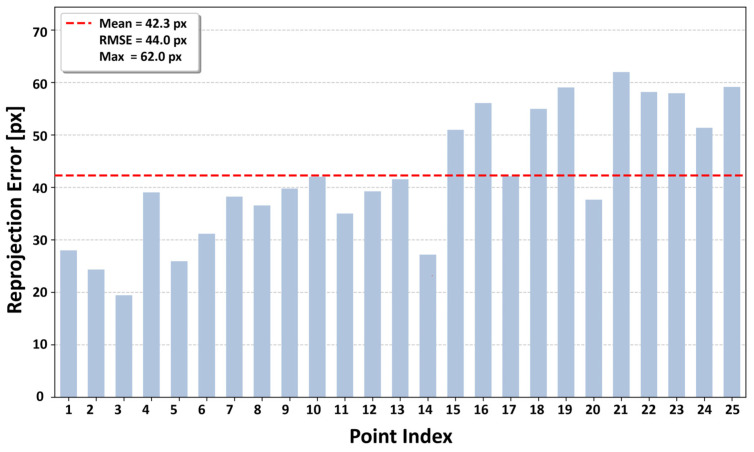
Reprojection errors for 25 representative image–point correspondences.

To quantitatively assess correspondence accuracy, we selected 25 distinctive image points distributed from the center to peripheral regions and computed the pixel distances between their observed locations compared to the projected locations obtained from the corresponding 3D points using the estimated parameters. Figure 8 reports the reprojection error for each correspondence point; the dashed line indicates the mean error. The mean reprojection error was 42.3 pixels, the root mean square error (RMSE) was 44.0 pixels, and the maximum error was 62.0 pixels.

### 4.2. Evaluation Metrics

To quantitatively assess noise removal performance, we evaluated precision, recall, and intersection over union (IoU). Each metric is computed by comparing the system’s successfully detected noise points (positives) with manually annotated ground-truth labels, following Equations (4)–(6), respectively. Points correctly detected as human-related noise are counted as true positives (TPs); background points (e.g., walls) incorrectly detected as noise are false positives (FPs); and human-related noise that the system failed to detect are false negatives (FN).

(4) $$Precision=\frac{TP}{TP+FP}$$

(5) $$Recall=\frac{TP}{TP+FN}$$

(6) $$IoU=\frac{TP}{TP+FP+FN}$$

### 4.3. Parameter Validation

#### 4.3.1. Parameter Settings for DBSCAN and Radius Parameter in the Cylindrical Filter

To investigate the optimal parameter settings for human extraction in the proposed method, we conducted a grid search using the DBSCAN distance threshold $\varepsilon \;\left(eps\right)$ and the cylinder filter radius r as variables. Because DBSCAN is applied in 3D coordinate space (dim = 3), we set min_samples (MinPts) to 6 following the common rule-of-thumb MinPts ≈ 2 × dim [5]. For this analysis, the planarity threshold P, which serves as the criterion for background removal, was fixed at 0.6. This setting is based on the results of the parameter sensitivity analysis detailed in Section 4.3.2. By fixing the geometric criterion based on these preliminary findings, this section focuses on evaluating the impact of the clustering and cylinder filtering parameters on the overall accuracy.

(i) Performance Distribution Comparison Using IoU Heatmaps

**Figure 9:**
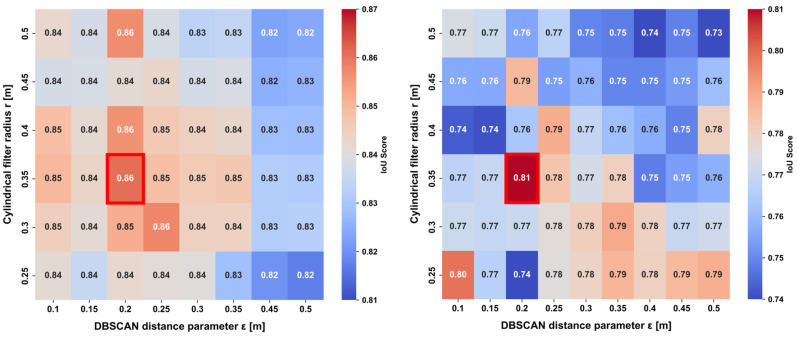
Comparison of intersection over union (IoU) performance heatmaps P=0.60. (**Left**): Heatmap analysis results for Osaka Metropolitan University, Sugimoto Campus. (**Right**): Heatmap analysis results for Jinaimachi, Tondabayashi City, Osaka Prefecture. (Red squares indicate the optimal thresholds ε = 0.25 and r = 0.35).

Figure 9 presents the IoU results for the two datasets in the form of heatmaps. To clearly visualize the relative performance distribution within each dataset, the color bars of the heatmaps are individually normalized based on the minimum and maximum IoU values of each dataset (local scaling). This design choice is motivated by the observation that the Osaka Metropolitan University, Sugimoto Campus, dataset exhibits consistently high IoU values overall (0.81–0.87), whereas the Jinaimachi dataset in Tondabayashi City shows a wider spread of sensitivity across parameter settings (0.73–0.81). Under a shared color scale, subtle variations in performance within each environment would be difficult to discern.

Therefore, absolute performance comparisons between the datasets are discussed separately based on the quantitative evaluation results summarized in Table 1.

(ii) Optimal Parameter Selection

Although the overall performance distributions exhibit low correlation between the two datasets, the parameter set that achieved the maximum IoU was identical for both datasets, namely ε=0.25 m and r=0.35 m. The key performance metrics obtained under this optimal parameter setting are summarized in Table 1.

**Figure 10:**
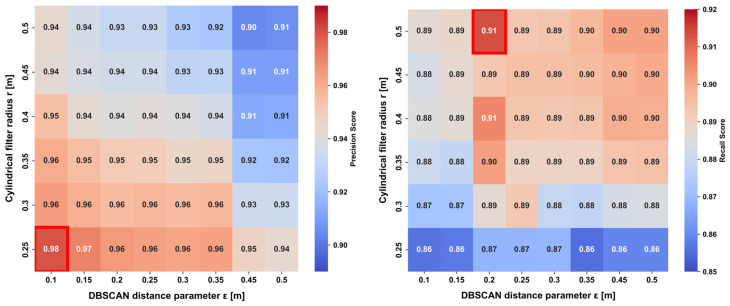
Trade-off between precision and recall (Osaka Metropolitan University, Sugimoto Campus). (**Left**): Precision heatmap. (**Right**): Recall heatmap.

The fact that the optimal parameter values are consistent across two distinct environments suggests that the proposed method effectively captures the intrinsic spatial density structure of human point clouds, rather than being sensitive to environment-dependent variations. Furthermore, an analysis of the precision and recall heatmaps shown in Figure 10 (presented using the OMU dataset as a representative example) reveals that, around ε=0.20~0.25 m, both point-cloud over-segmentation (leading to decreased recall) and erroneous noise aggregation (leading to decreased precision) are simultaneously minimized. This results in an optimal trade-off between the two metrics. This observation indicates that, in human extraction tasks, a distance threshold of ε=0.20~0.25 m functions as a geometric “critical threshold” for clustering, effectively balancing the continuity of the human body with its separation from background structures.

(iii) Universal Robust Region

To examine the flexibility of parameter settings in practical applications, we define a robust region as the parameter space in which the IoU values fall within the top 25% for both datasets. This criterion is designed to identify parameter ranges that ensure stable performance with IoU values exceeding 0.80 across diverse environments.

As a result, it was found that consistently high performance is maintained within the following parameter ranges, independent of environmental differences.

- Neighborhood search radius ε for DBSCAN clustering: 0.20 ≤ϵ≤0.25;
- Cylinder radius r: 0.35 ≤r≤0.40.

These results suggest that, when applying the proposed method to previously unseen environments, initializing the parameters within the above range is highly effective for ensuring stable performance. Although environment-specific sensitivity differences are observed, the existence of a shared high-performance region at the center of the parameter space provides important evidence of the practical robustness of the proposed method.

Accordingly, for the subsequent experiments and evaluations presented in this study, the parameter values ε=0.25 m and r=0.35 m are adopted.

#### 4.3.2. Parameter Settings for Principal Component Analysis

**Figure 11:**
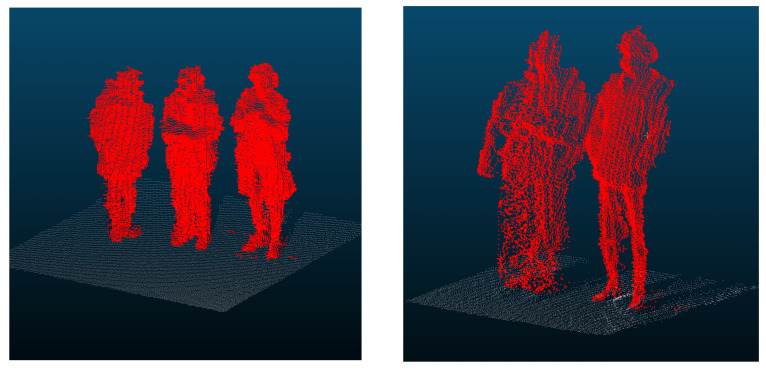
Examples of point-cloud data used for geometric feature evaluation. Side-by-side humans, which are likely to be misrecognized as a wall.

To design a high-accuracy filter capable of distinguishing humans from wall surfaces, we first quantitatively evaluated the differences in their local geometric features. Drawing on the examination of distance-dependent point counts discussed in Section 4.3.3, we divided the observation range into short-range (<5 m), mid-range (5–20 m), and long-range (≥20 m) categories, thereby ensuring robust threshold settings. We prepared an evaluation dataset consisting of 30 labeled samples in total (15 walls and 15 humans), with 5 samples selected from each distance category. Notably, the human samples intentionally included patterns of multiple individuals standing side-by-side, a configuration that is particularly prone to being misidentified as a wall. Figure 11 illustrates examples of these side-by-side human and wall-surface point clouds used for evaluation. For each point in these clouds, we applied principal component analysis (PCA) to its neighborhood and computed the planarity score from the eigenvalues of the covariance matrix (Equation (7)). This score approaches 1 for planar structures and 0 for volumetric (non-planar) structures.

(7) $$P=\frac{\lambda_{2}-\lambda_{3}}{\lambda_{1}}\;\left(\lambda_{1}\geq \lambda_{2}\geq \lambda_{3}\right)$$

(i) Stability Evaluation with Respect to Observation Distance and Point-Cloud Density

To determine the planarity score threshold used in the proposed method, we conducted a stability analysis using 30 prepared samples. To synthetically reproduce variations in point-cloud density, each sample was subjected to five density levels (100%, 80%, 60%, 40%, and 20%). In addition, to eliminate fluctuations caused by the stochastic behavior of the RANSAC algorithm, five independent random sampling trials were performed under identical conditions.

**Figure 12:**
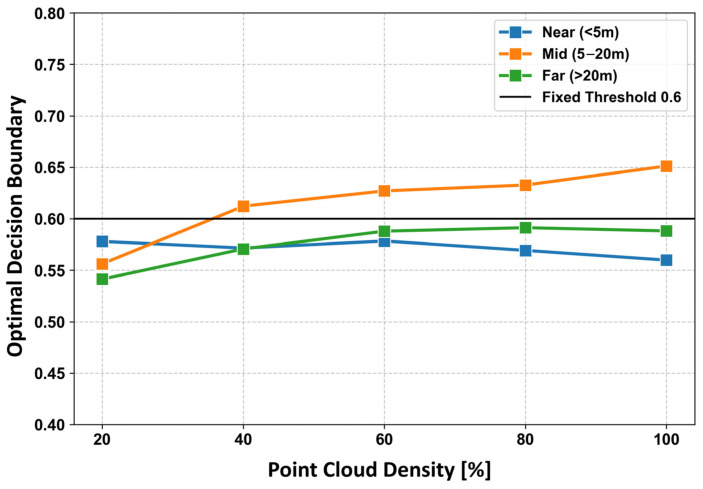
Transition of planarity score decision boundaries across various observation distances and point cloud densities.

As a result, a total of 750 experimental cases (30 files × 5 density levels × 5 trials) were generated, ensuring sufficient statistical reliability. Figure 12 illustrates the intersection points of the score distributions for humans and wall surfaces, estimated using kernel density estimation (KDE), and how these transition with different observation distances and density conditions. The horizontal axis represents the point-cloud density after downsampling (%), while the vertical axis indicates the boundary score between the two classes derived from KDE under each condition.

In certain conditions—such as long observation distances combined with low point-cloud density—the score distributions for humans and walls were completely separated, making it mathematically impossible to compute a KDE-based intersection. In these cases, the midpoint between the two distributions was adopted as the boundary score in order to maintain statistical continuity across conditions. Figure 12 clearly shows that the intersection values are generally concentrated within the range of 0.55 to 0.60. Based on this observation, the validation in Section 3.4.3 adopts this statistically derived boundary, and the planarity score threshold is set to 0.6. It is worth noting that, particularly under the long-distance and 20% density condition, the intersection value decreases to approximately 0.532. This trend can be attributed to two factors: the reduction in point-cloud density causes a decrease in the score for humans and wall surfaces—which are inherently planar—and also exhibit a significant drop in PCA-based planarity scores due to the limited number of points. Consequently, the entire score distribution shifts toward lower values.

**Figure 13:**
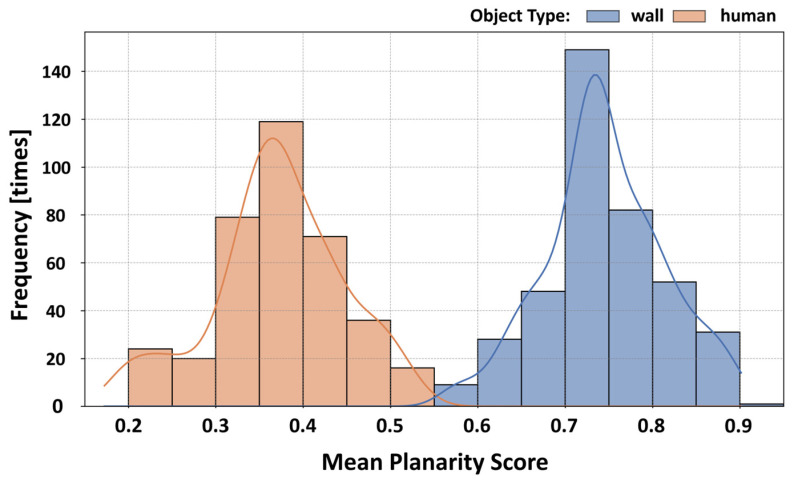
Aggregated frequency distribution and kernel density estimation (KDE) curves of planarity scores for all experimental conditions.

To comprehensively evaluate these detailed distribution characteristics, Figure 13 presents the aggregated distribution of the mean planarity scores across all 750 samples. As shown by the histogram and KDE curves in Figure 13, even when all conditions are combined, the score distributions of humans and walls remain clearly separated. In addition, the intersection consistently lies around 0.55–0.60.

These results demonstrate that the fixed threshold of 0.6 adopted in this study exhibits strong robustness against variations in measurement conditions, including observation distance and point-cloud density.

(ii) Threshold Sensitivity Analysis and Final Optimization

To determine the final parameter setting that governs the overall system performance, we evaluated the results obtained by sweeping the planarity score threshold, P, from 0.40 to 0.80 in increments of 0.05. The DBSCAN distance threshold was fixed at ε=0.25 m, and the cylindrical filter radius was r=0.35 m. These values were identified as optimal in Section 4.3.1.

As shown in Table 2, the OMU dataset achieved its maximum IoU of 0.8607 at P=0.6. In contrast, the JM dataset exhibited its highest IoU of 0.8186 at P=0.55; however, the IoU at P=0.6 was 0.8132, which is extremely close, with a difference of only 0.005 (approximately 0.5%).

As the planarity score threshold, P, increases, fewer human points are mistakenly classified as planar surfaces and removed, resulting in an overall improvement in recall for both datasets. However, when P exceeds 0.60, boundary noise from wall surfaces begins to erroneously merge into human clusters. This behavior disrupts the balance between precision and recall, leading to saturation or degradation of IoU.

Considering these results together with the geometric robustness analysis described in Section 4.3.2 (i), it is evident that P=0.6 represents a universal optimal threshold that consistently maximizes human extraction performance across different outdoor environments. This convergence indicates that the proposed method provides a highly practical threshold setting for separating humans from TLS point clouds, without requiring environment-specific parameter tuning.

#### 4.3.3. Distance Threshold Setting

In this study, a horizontal distance of 30 m from the scanner was defined as the effective processing range. This threshold was determined by jointly considering (i) the physical characteristics of the device, (ii) the spatial distribution of the point-cloud data, and (iii) detection reliability in real-world environments.

First, the accuracy range of the Matterport Pro3 scanner used in this study was approximately ±20 mm at a distance of 10 m, and both measurement error and point-cloud sparsity inevitably increased as the measurement distance grew. To quantify the spatial distribution of the acquired data, a statistical analysis was conducted using 50 randomly selected scans. The results showed that, out of an average of approximately 3.33 million points per scan—a figure significantly exceeding the standard yield of 1.5 million points due to the open outdoor environment—only 3.44% (approximately 115,000 points) were located beyond a distance of 30 m. Moreover, these distant points mainly correspond to large static structures such as walls and buildings, while the number of points belonging to comparatively small human objects is extremely limited. This indicates that point-cloud data beyond 30 m contributes little to overall scene reconstruction, while simultaneously exhibiting higher uncertainty as noise due to low density.

Next, to evaluate the stability of human detection with respect to distance, a controlled experiment was conducted in which human subjects were placed at distances ranging from 10 m to 35 m at intervals of 5 m. The results demonstrated that detection remained highly stable up to 20 m, with the average IoU exceeding 0.96. However, detection performance gradually degraded with increasing distance: four out of five subjects were successfully detected at 25 m, and three out of five were detected at 30 m. At 35 m, detection performance deteriorated markedly, with only one out of five subjects successfully detected, primarily due to severe point-cloud sparsity and segmentation failures.

Based on these results, 30 m was identified as the optimal distance threshold to ensure reliable noise removal based on 2D segmentation while preserving more than 96% of the total point-cloud data to maintain global scene consistency. By focusing processing on the near-range region where point-cloud quality is high, the proposed system achieves a balance between computational efficiency and detection accuracy.

#### 4.3.4. Choice of 2D Model (Ablation)

To select the 2D segmentation model that would serve as the foundation of the proposed method, we conducted a comparative evaluation of candidate models. Table 3 and Table 4 summarize the detection accuracy (precision, recall, and IoU) and computational performance (FPS and total processing time) for each model.

The results indicate that YOLOv8, with the standard input resolution, achieved superior performance across both datasets, attaining high IoU scores (OMU: 0.8607, JM: 0.8132) and maintaining a favorable processing speed (3.89 FPS) and shorter total processing time. In contrast, tile-based variants (YOLOv8 (tiling) and YOLOv12 (tiling)) processed 4096 × 4096 images by dividing them into smaller patches. This strategy improves memory locality but increases runtime due to overlap and repeated inference.

In the proposed framework, erroneous detections caused by projection inaccuracies and background structures are systematically suppressed by three-dimensional geometric filtering (Section 3.4) applied after 2D segmentation. Owing to this design, highly accurate noise removal can be achieved using the outputs of the standard YOLOv8 model, without resorting to computationally expensive tile-based processing at the 2D stage.

Based on these findings, the standard YOLOv8 model, which offers the best balance between computational efficiency and detection accuracy, was selected as the 2D inference engine for the proposed system.

### 4.4. Overall Evaluation Setup and Baseline Definition

To assess the contribution of each stage in the proposed geometric filtering pipeline, an ablation study with five configurations has been conducted:

(1) Simple projection of 2D segmentation results (2D-only);
(2) Addition of 3D clustering;
(3) Addition of cylindrical processing;
(4) Addition of background segmentation;
(5) The full proposed method with cluster-level human plausibility validation (proposed).

**Figure 14:**
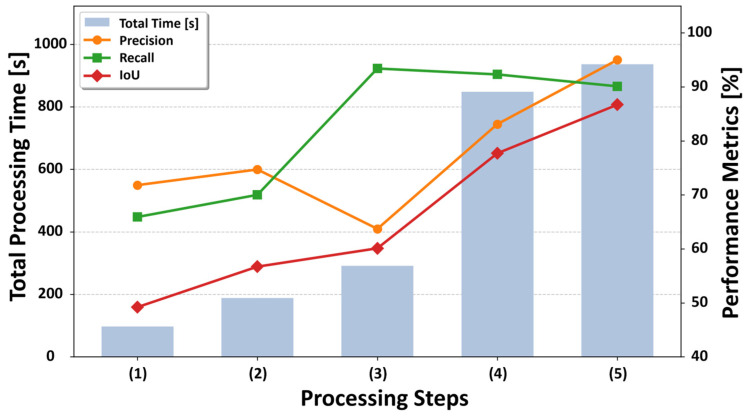
Evaluation results for the Osaka Metropolitan University, Sugimoto Campus dataset.

**Figure 15:**
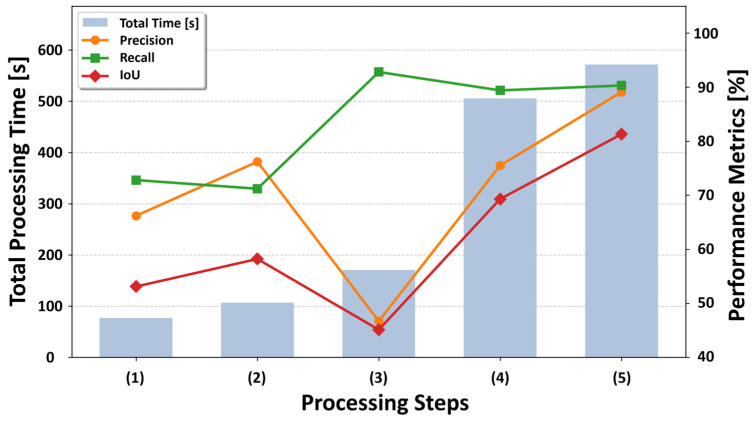
Evaluation results for the Tondabayashi City, Osaka Prefecture dataset.

We evaluate all configurations on the OMU and JM datasets using precision, recall, and IoU, while also recording the processing time for each stage. The quantitative ablation results and computational cost are reported in Section 5.1 (Table 5 and Figure 14 and Figure 15).

## 5. Results

### 5.1. Ablation Study Results and Computational Cost

To clarify how each component contributes to end-to-end noise removal, we report an ablation study in which processing stages are incrementally added from (1) to (5). Table 5 summarizes the quantitative performance (precision/recall/IoU) and cumulative processing time for both datasets; Figure 14 and Figure 15 visualize the corresponding evaluation results. To clarify the computational breakdown, we calculated the average incremental processing time across the two datasets for each stage. The specific costs were 87.0 s for (1) simple projection of 2D segmentation results (including 2D inference); 60.3 s for (2) addition of 3D clustering; 83.5 s for (3) addition of cylindrical processing; 446.0 s for (4) addition of background segmentation; and 76.9 s for (5) cluster-level human plausibility validation.

Table 5 summarizes how the proposed pipeline resolves the precision–recall trade-off as stages (1)–(5) are performed. Compared to stage (2), cylindrical processing in stage (3) markedly increases recall by geometrically re-incorporating partially missed limbs around the projected human region (OMU: 0.7001 → 0.9340; JM: 0.7117 → 0.9283). This expansion intentionally prioritizes coverage of human-related noise, but it can also include nearby background surfaces, leading to a temporary drop in precision (OMU: 0.7469 → 0.6373; JM: 0.7617 → 0.4671). In stage (4), background segmentation suppresses these false positives based on 3D geometric characteristics, recovering precision (OMU: 0.8310; JM: 0.7546) while maintaining high recall (OMU: 0.9230; JM: 0.8941), which yields a strong increase in IoU (OMU: 0.7771; JM: 0.6927). Finally, the full pipeline in stage (5) achieves the best overall balance, reaching the highest precision (OMU: 0.9502; JM: 0.8912) and peak IoU (OMU: 0.8607; JM: 0.8132). From an efficiency standpoint, background segmentation is the most computationally demanding step (incremental ≈ 557 s for OMU and ≈335 s for JM). This translates to an average processing time of approximately 11.4 s per scan for the OMU dataset and 8.4 s per scan for the JM dataset, confirming the system’s practical runtime feasibility. However, it is also the key contributor to precision recovery and IoU improvement; moreover, any residual 2D–3D misalignment is effectively mitigated by the subsequent geometric filtering and cluster-level validation. Figure 14 and Figure 15 visualize these trends, highlighting how geometric gating resolves the initial recall-oriented expansion into high-precision removal.

This behavior is quantitatively supported by the robust performance of the full pipeline (stage 5) shown in Table 5. However, the objective of the proposed method is not pixel-level precise correspondence, but rather stable removal of three-dimensional noise caused by minor subject motion and/or reprojection misalignment in real-world environments. In the proposed pipeline, local mismatches and isolated points arising from reprojection errors are effectively suppressed by the small-noise-cluster removal process described in Section 3.4.1. Furthermore, the geometric processing steps in Section 3.4.4 perform cluster-level plausibility checks, enabling reliable removal of non-human regions even in the presence of projection inaccuracies. Overall, the results in Section 5.1 indicate that the range of reprojection errors observed in this study does not undermine the practicality or effectiveness of the proposed approach.

### 5.2. Quantitative Results on Two Real-World Datasets

#### 5.2.1. Overall Performance of the Proposed Method

Figure 14 and Figure 15 summarize the quantitative performance of the five ablation settings on the two datasets. Compared with the simple projection of 2D segmentation results (the 2D-only method (1)), the full proposed method incorporating human-based validation (the proposed method (5)) achieves the highest overall precision and IoU for both datasets. These results demonstrate that the proposed method operates as a role-aware, multi-stage pipeline in which intermediate trade-offs are intentionally introduced and subsequently resolved, resulting in robust overall detection performance across different environments.

#### 5.2.2. Comparison with Geometry-Based Sequential Dynamic Object Removal Methods

In this section, we compare the performance of the proposed method, which integrates 2D–3D semantic information based on YOLOv8, with DUFOMap and BeautyMap, which are representative time-series geometric dynamic object removal methods designed for multi-pass or sequential LiDAR mapping scenarios with repeated observations. The evaluation is conducted using two real-world TLS datasets, OMU and JM, which differ substantially in their acquisition environments and structural characteristics. These datasets serve as complementary test cases for analyzing the applicability and limitations of general-purpose dynamic object removal methods and the proposed human-noise-oriented approach. DUFOMap and BeautyMap are designed to remove general dynamic objects, including not only humans but also vehicles and moving vegetation. In this study, these methods are evaluated under static TLS acquisition constraints to clarify potential assumption gaps with respect to limited revisit frequency and viewpoint diversity. However, under the ground-truth (GT) definition used in this study—which contains only human-induced noise—the performance of these methods is most appropriately assessed in terms of recall, as it directly reflects their ability to detect and remove human points. Accordingly, recall is adopted as the primary evaluation metric for DUFOMap and BeautyMap. To ensure fairness and reproducibility, all experiments for these methods were conducted using the recommended parameter settings provided by the original implementations, without any dataset-specific tuning. In contrast, since the proposed method is specifically designed for human noise removal, we additionally report precision and intersection over union (IoU), enabling an evaluation of both accurate human-region preservation and the suppression of erroneous removal of non-human structures.

(i) Overview of Quantitative Results

The quantitative evaluation results for the OMU and JM datasets are summarized in Table 6 and Table 7. For each dataset, we report recall, precision, intersection over union (IoU), and total processing time. Because processing time strongly depends on implementation details and computational environments, it is not intended as a strict measure of algorithmic complexity, but rather as an indicator of relative tendencies arising from dataset characteristics and acquisition conditions. Note that the set of reported metrics in Table 6 and Table 7 is determined based on the design objectives of each method and the definition of the ground truth (GT).

(ii) Results from the OMU Dataset

With the OMU dataset, DUFOMap achieved a high recall of 0.9316. The proposed method exhibited a slightly lower recall of 0.9014; however, unlike general-purpose dynamic object removal methods, it additionally enables the evaluation of precision and IoU, achieving a precision of 0.9502 and an IoU of 0.8607 under the human-only GT definition. A quantitative analysis of the height distribution of false negatives (FN) revealed that, for DUFOMap, FN points were strongly concentrated near the ground surface. Specifically, more than 65% of all FN points were located within 0.3 m above ground, with a median FN height of 0.214 m, indicating that missed removals predominantly occurred in foot-level regions, which are particularly challenging for time-series geometric methods under TLS acquisition conditions. FN points were also observed in sparsely sampled regions such as the torso and head, suggesting sensitivity to partial observations and uneven point density.

**Figure 16:**
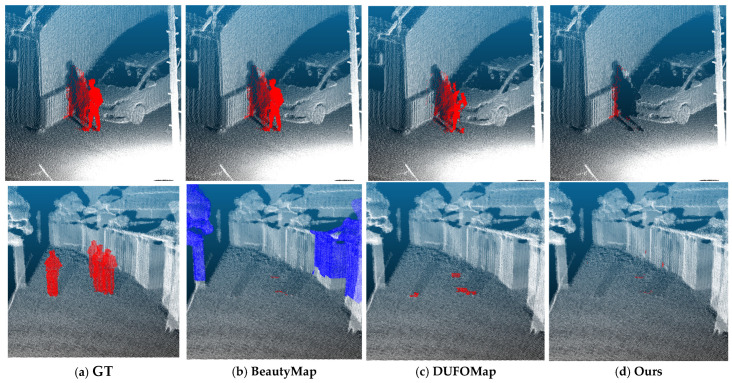
Qualitative comparison of BeautyMap, DUFOMap, and the proposed method on the JM dataset. Top row: Standing human observed in a single scan near a wall. (**a**) Original point cloud, where the target human is highlighted in red. (**b**) BeautyMap result, where the human is not detected (no effective human removal is observed in this case). (**c**) DUFOMap result, in which the human remains due to single-scan observation and occlusion. (**d**) Result of the proposed method, where the target human is successfully removed. Bottom row: Standing human with foot-level absorption near the ground. (**a**) Original point cloud with the target human highlighted in red. (**b**) BeautyMap result, where foot-level human points are successfully detected; however, part of the nearby wall is mistakenly removed, shown in blue (false removal of static background). (**c**) DUFOMap result, where foot-level human points are merged into ground voxels, leading to missed removal. (**d**) Result of the proposed method, where the entire human region is removed. Red points indicate human points of interest, and blue points indicate erroneously removed static background points.

For BeautyMap, the recall on the OMU dataset was 0.7343. While BeautyMap exhibited relatively fewer missed removals in foot-level regions compared to DUFOMap, it tended to misclassify static structures—such as building facades and surrounding architectural elements—as dynamic objects under the evaluated TLS conditions, resulting in excessive removal of structures that should have been preserved (Figure 16, bottom row (b)). These behaviors can be largely attributed to the reliance on geometric and temporal consistency across multiple observations, combined with ray occlusions and limited observation redundancy under typical TLS acquisition settings. Ray-casting-based probabilistic updates and grid-based occupancy reconstruction are vulnerable to occlusions and insufficient observation redundancy, particularly under TLS conditions characterized by wide scan spacing and partial observations, which increases the likelihood of misclassification.

In contrast, the proposed method projects human semantic information obtained from 2D images into 3D space and preserves each person as a consistent semantic entity. As a result, it is less affected by local point-density variations, ray occlusions, and geometric absorption into the ground, enabling stable extraction of human regions even under challenging TLS conditions.

(iii) Results on the JM Dataset

On the JM dataset, the recall of DUFOMap dropped significantly to 0.6479, whereas the proposed method maintained a high recall of 0.9028, resulting in a pronounced gap in recall performance. In terms of processing time, the proposed method also completed the evaluation substantially faster than DUFOMap (571.58 s vs. 1842.68 s), indicating differences in computational cost under complex urban conditions. For BeautyMap, the recall on the JM dataset was 0.5226, with a processing time of 4936.39 s, indicating a substantial degradation in both detection performance and computational efficiency. When using the default settings recommended for outdoor environments, BeautyMap exhibited a “skip” phenomenon, in which processing was not executed for certain scans due to the wide spacing between TLS scans, leading to reduced recall. Expanding the search range mitigated the skip phenomenon and partially improved recall, at the cost of increased erroneous removal of static structures. However, this improvement was accompanied by a clear increase in erroneous removal of static structures, resulting in a substantial degradation of structural preservation. The results reported in Table 6 and Table 7 therefore include configurations in which a trade-off between recall improvement and increased false removals becomes evident.

The JM dataset represents a dense urban environment composed of roads and closely packed buildings, featuring numerous structural occlusions such as walls, corners, and garage surroundings. Under such conditions, TLS scans are widely spaced while each individual scan remains highly dense, resulting in a limited number of viewpoints. Consequently, many pedestrians are observed only temporarily, producing a large number of single-scan observations. Because DUFOMap relies on temporal and geometric consistency across multiple viewpoints, such transiently observed individuals are prone to being misclassified as static objects, leading to increased false negatives.

Furthermore, when scan spacing is large, even static structures may lack sufficient observation redundancy. Expanding the observation range integrates distant points with reduced point density and geometric accuracy, increasing the likelihood of dynamic classification based on unreliable geometric evidence. Under these conditions, BeautyMap becomes prone to excessive removal of static structures, misclassifying them as dynamic objects.

(iv) Qualitative Comparison and Failure Analysis

Figure 16 presents a qualitative comparison of DUFOMap, BeautyMap, and the proposed method on the JM dataset. Overall, the results on the OMU and JM datasets suggest that DUFOMap and BeautyMap exhibit complementary failure characteristics under TLS acquisition conditions. DUFOMap tends to produce under-removal, particularly in foot-level regions and sparsely sampled body parts, due to insufficient temporal observation evidence. In contrast, while BeautyMap can locally reduce foot-level false negatives, it suffers from substantial degradation in recall performance and computational cost on the JM dataset, accompanied by pronounced over-removal of static structures under conditions of limited observation redundancy. These findings indicate that, under TLS acquisition characteristics—namely wide scan spacing combined with high per-scan point density—simply expanding spatial or temporal integration ranges does not consistently resolve these issues.

In contrast, the proposed method preserves humans as semantic entities within a single scan and applies geometry-aware validation after projection, thereby reducing dependence on observation frequency and geometric statistics. As a result, it achieves stable and consistent human noise removal across diverse TLS conditions, including dense urban street environments.

This comparison highlights fundamental differences between time-series geometric approaches and semantic-driven single-scan processing for TLS-based human noise removal.

### 5.3. Generalization to MMS Data

The evaluation on the Waymo Open Dataset achieved Precision = 0.921, Recall = 0.814, and IoU = 0.761. Although these values are slightly lower than those obtained on the TLS datasets (Section 5.1 and Section 5.2.1), the proposed method maintained a stable level of performance without parameter recalibration, indicating that the TLS-optimized configuration can be applied without modification in the evaluated MMS dataset. A detailed inspection of the degraded cases revealed several representative failure patterns observed in our experiments, as summarized below.

(1) Degradation under low-contrast conditions

In outdoor scenes where strong sunlight produces deep shadows under trees, pedestrians wearing dark-colored clothing were occasionally missed by YOLOv8. In such cases, the luminance contrast between the targets and the background becomes weak, resulting in unstable 2D segmentation.

(2) Occlusion by nearby structures

The proposed pipeline assigns a detected person by selecting, for each 2D detection region, the corresponding 3D cluster that is closest to the camera position. This design effectively suppresses false positives caused by background structures located behind pedestrians. However, when thin structures such as poles or streetlights are located directly in front of pedestrians and are included in the 2D detection region due to projection errors, the foreground structures may be mistakenly selected as human clusters. Such misclassifications were observed not only in MMS data but also in TLS datasets under similar occlusion conditions.

These findings indicate that our TLS-optimized pipeline exhibits stable performance on the evaluated MMS dataset without further tuning, achieving reliable human extraction under dynamic mobile mapping conditions.

### 5.4. Qualitative Visualization of Human Extraction and Removal

**Figure 17:**
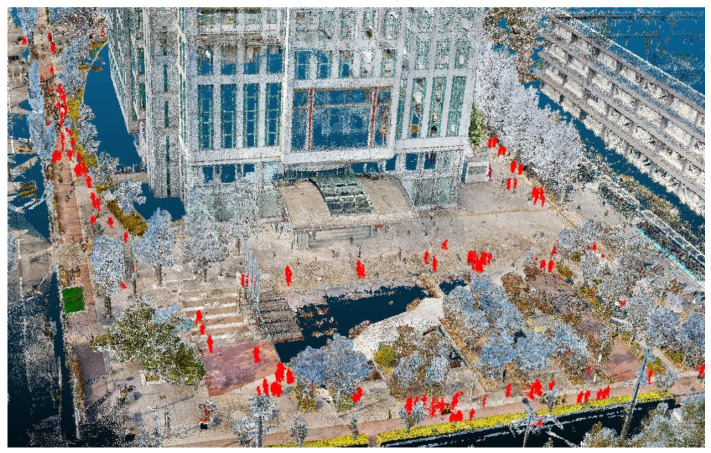
Classification results on the Sugimoto Campus dataset using the proposed method (humans are shown in red).

**Figure 18:**
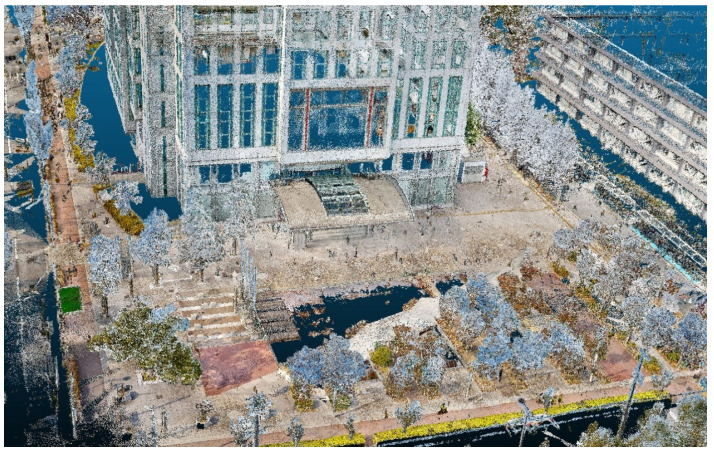
Final point cloud after removing human points from the Sugimoto Campus dataset (proposed method).

To visually demonstrate the end-to-end effect of the proposed method, Figure 17 presents the classification results applied to the entire Sugimoto Campus dataset of Osaka Metropolitan University, and Figure 18 shows the final output after removing the human point clouds. From Figure 17, we can confirm that the points classified as humans (shown in red) are correctly extracted. From Figure 18, we observe that the human points identified by the classification stage are effectively removed without encroaching on surrounding background structures such as buildings and vegetation.

## 6. Discussion

Small 2D–3D misalignments in image-to-point-cloud projection can cause background points behind target objects to be falsely included, which is a major source of precision degradation in 2D-only pipelines. The proposed method addresses this issue by introducing projection-followed, geometry-aware validation (Section 3.4), which progressively transforms an initially recall-oriented candidate expansion into high-precision noise removal. As demonstrated in the ablation study (Section 5.1 and Table 5), the cylindrical filtering stage intentionally increases recall by re-incorporating partially missing limb points, while subsequent background segmentation and cluster-level plausibility checks effectively recover precision and maximize IoU. The observation that similar behavior is preserved on MMS data further supports the general applicability of this multi-stage geometric gating strategy.

Beyond overall performance metrics, the comparison with time-series-based geometric dynamic object removal methods, including DUFOMap, provides important insight into characteristic failure mechanisms under TLS acquisition conditions. On the OMU dataset, DUFOMap achieved a high recall; however, a detailed analysis revealed that more than 65% of its false negatives (FN) were concentrated within 0.3 m above the ground, with a median FN height of 0.214 m. This indicates that missed detections predominantly occur in foot-level regions, even for standing pedestrians. These failures are closely related to the reliance on ray-casting-based probabilistic updates and occupancy reasoning, under which observations near the ground surface are highly susceptible to occlusion and therefore fail to accumulate sufficient evidence for dynamic classification. Moreover, the wide scan spacing inherent to TLS, combined with partial occlusions, limits the availability of repeated observations, particularly for lower-body regions, thereby restricting the statistical evidence available for reliable dynamic inference.

Importantly, these failure modes are not merely attributable to suboptimal parameter settings but are fundamentally linked to TLS-specific acquisition characteristics, including wide scan intervals and frequent structural occlusions. This tendency becomes more pronounced in complex urban environments such as the JM dataset, where pedestrians are often observed in only a single scan due to walls, corners, or roadside structures. Under such conditions, the assumption of repeated observations across multiple viewpoints—central to time-series–based geometric reasoning—is frequently violated, leading to systematic detection failures. Similar issues arise when attempting to compensate for insufficient temporal observations by expanding the spatial integration range. In fact, for BeautyMap, our experiments under TLS conditions showed that such configurations led to pronounced over-removal of static structures, where buildings and surrounding architectural elements were misclassified as dynamic objects. This outcome suggests that, in time-series-based geometric approaches, strategies that seek to address limited observation redundancy through spatial expansion alone can introduce alternative forms of misclassification.

In contrast, the proposed method is explicitly designed to operate under these TLS-specific constraints by leveraging semantic information derived from 2D images to preserve the entire human body as a consistent entity within a single scan. This design choice reduces reliance on observation frequency and purely geometric evidence, explaining the smaller performance variation observed across datasets with markedly different structural complexity, such as OMU and the densely occluded JM environment.

Our reprojection analysis reported a moderate error level (RMSE ≈ 44 px; Section 4.1.4). While reducing this error is desirable, the objective of the proposed system is not pixel-level correspondence but stable suppression of human-related noise under real-world acquisition conditions. Local mismatches and isolated points induced by projection inaccuracies are effectively suppressed through small-cluster removal (Section 3.4.1) and subsequent cluster-level validation (Section 3.4.4). The consistently stable Precision, Recall, and IoU observed in the final stage indicate that the measured reprojection error range does not compromise the practical reliability of the pipeline. This geometric gating is particularly important for preventing unintended removal of nearby static structures (e.g., thin poles or furniture) when residual calibration error causes slight offsets around human boundaries.

Performance degradation observed under low-contrast conditions in MMS data originates primarily from instability in the 2D image recognition stage rather than from LiDAR sensing itself. Under such conditions, image enhancement and restoration techniques, such as Variational single nighttime image Dehazing (VNDHR) [36], may serve as effective preprocessing steps to improve the robustness of 2D segmentation. Recent projection-based deep learning approaches, including Enhanced Semantic Segmentation of LiDAR Point Clouds Using Projection-Based Deep Learning Networks [37], aim to more tightly integrate image and point-cloud features and reduce the impact of projection errors and occlusions. However, these methods typically involve higher computational cost and stronger dependence on training data, which may limit their applicability in lightweight TLS-oriented pipelines.

**Figure 19:**
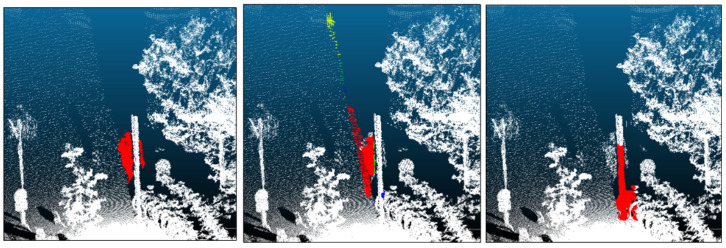
An example of a failure case (false negative) due to challenging conditions such as occlusion. (**Left**): Ground-truth human annotation (red). (**Center**): Intermediate clustering result (yellow/green/blue indicate different intermediate clusters; colors are for visualization). (**Right**): Final result showing a missed detection (false negative).

From an efficiency perspective, we compared multiple 2D instance segmentation configurations, including tile-based inference and Mask R-CNN, and confirmed that standard YOLOv8-seg offers a favorable speed–accuracy trade-off for the proposed pipeline (Section 4.3.4). While background segmentation is the most time-consuming stage, it is also the principal contributor to precision recovery and overall IoU improvement (Section 5.1). Finally, typical failure cases were observed under challenging conditions such as strong mutual occlusions (Figure 19), where both 2D masks and geometric cues become less reliable. Addressing these cases, along with broader benchmarking against dedicated 3D-only noise removal baselines, is important for future work.

In addition to the failure cases illustrated above, two limitations inherent to projection-based approaches should be noted.

First, in highly crowded pedestrian environments, strong mutual occlusions between individuals may lead to fragmented 2D instance masks, which in turn result in discontinuous or partially missing 3D point-cloud masks after projection. While the proposed multi-stage geometric validation mitigates small fragments through cluster-level plausibility checks, extreme crowding remains challenging due to the ambiguity of visual separation at the image level.

Second, the accuracy of 2D–3D mapping is inherently sensitive to the calibration between the TLS scanner and the camera system. Even small calibration errors can induce geometric misalignment, potentially causing thin static background structures—such as poles, railings, or furniture located near human subjects—to be mistakenly included in the removal region. In the proposed pipeline, this risk is partially alleviated by subsequent geometric filtering and background segmentation; however, accurate sensor calibration remains a critical prerequisite for reliable deployment.

These limitations are not unique to the proposed method but are common to projection-based frameworks that rely on cross-modal alignment. Addressing them through more robust calibration procedures, uncertainty-aware projection, or tighter image–geometry fusion constitutes an important direction for future work.

## 7. Conclusions

This study presented a geometry-aware framework for automatic and high-accuracy human noise removal from terrestrial laser scanning (TLS) point clouds. The proposed method integrates 2D instance segmentation using YOLOv8 with projection of semantic masks into 3D space, followed by multi-stage geometric validation based on PCA-driven filtering. The core contribution lies in the projection-followed geometric gating strategy, which effectively suppresses false positives caused by 2D–3D misalignment and clustering artifacts, without requiring any training of 3D deep neural networks.

Extensive experiments on two real-world TLS datasets demonstrated that the proposed pipeline achieves a stable balance between precision and recall and consistently improves IoU compared with 2D-only projection-based baselines. The ablation study clarified the role of each processing stage, showing that recall-oriented candidate expansion can be reliably transformed into high-precision human noise removal through background segmentation and cluster-level plausibility validation. Parameter sensitivity and robustness analyses further provided practical guidance for deploying the method under diverse TLS acquisition conditions, including variations in point density, observation distance, and scene structure.

Comparative analysis with state-of-the-art geometric methods revealed fundamental differences in design assumptions. Time-series–based approaches rely on repeated observations across multiple viewpoints and therefore tend to degrade under TLS-specific acquisition constraints, such as wide scan intervals and frequent occlusions commonly observed in urban environments. In contrast, the proposed method is explicitly designed to operate under these TLS-specific constraints by preserving human regions as consistent semantic entities within a single scan. This design choice explains the stable performance observed across structurally different scenes, including wall-adjacent pedestrians and corner regions frequently encountered in TLS-based urban mapping.

While the primary evaluation focused on TLS data, additional experiments on MMS data also demonstrated that the proposed method achieves a certain level of human noise removal performance. Nevertheless, compared with TLS, partial performance degradation was observed under specific conditions. Detailed analysis suggests that this degradation originates not from limitations of LiDAR sensing itself, but mainly from instability in the 2D image recognition stage under low-contrast environments. Therefore, improving robustness at the image recognition stage—through image enhancement, restoration techniques, or tighter 2D–3D integration—represents an important direction for further improving generalization performance across datasets with different sensing characteristics.

In future work, although this study primarily addressed human noise removal, the proposed framework can be extended to other dynamic objects such as vehicles or temporary structures. Further investigation is also required to enhance robustness under complex human poses, high-density crowds, and strong mutual occlusions. For reproducibility and future research, the manually annotated human noise labels used in this study have been publicly released. Broader benchmarking against dedicated 3D-only and hybrid noise removal methods on additional TLS and MMS datasets will help establish a more versatile and generalizable point-cloud noise removal framework.

## Figures and Tables

**Table 1:** Best performance metrics at common optimal parameters.

Dataset	Precision	Recall	IoU
OMU	0.9502	0.9014	0.8607
JM	0.8912	0.9028	0.8132

**Table 2:** Variation in IoU with respect to planarity score.

Planarity Score	Osaka Metropolitan University, Sugimoto Campus [IoU]	Jinaimachi, Tondabayashi City [IoU]
0.40	0.6044	0.785
0.45	0.7819	0.7953
0.50	0.8316	0.7798
0.55	0.8432	**0.8186**
0.60	**0.8607**	0.8132
0.65	0.8584	0.7721
0.70	0.8588	0.7739
0.75	0.8586	0.7939
0.80	0.8586	0.7894

Note: The maximum IoU value in each column is shown in bold.

**Table 3:** Comparison of FPS and detection performance across object detection models (Osaka Metropolitan University, Sugimoto Campus).

Model	FPS	Total Time [s]	Precision	Recall	IoU
YOLOv8	3.89	935.62	0.9502	0.9014	0.8607
YOLOv12	3.96	938.18	0.9444	0.8964	0.8515
YOLOv8 *	2.20	990.15	0.9470	0.8810	0.8396
YOLOv12 *	1.56	1029.07	0.9471	0.8834	0.8419
Mask R-CNN *	0.59	1663.09	0.9408	0.8897	0.8425

Note: Rows marked with * were processed using tiling: the 4096 × 4096 input image was divided into 25 tiles (1024 × 1024 pixels) with a 100-pixel overlap.

**Table 4:** Comparison of FPS and detection performance across object detection models (Jinaimachi, Tondabayashi City, Osaka Prefecture, Japan).

Model	FPS	Total Time [s]	Precision	Recall	IoU
YOLOv8	3.89	571.58	0.8912	0.9028	0.8132
YOLOv12	3.96	569.91	0.8549	0.8971	0.7786
YOLOv8 *	2.20	582.68	0.9112	0.8076	0.7486
YOLOv12 *	1.56	572.01	0.8823	0.9003	0.8038
Mask R-CNN *	0.59	689.17	0.9090	0.8152	0.7537

Note: Rows marked with * were processed using tiling: the 4096 × 4096 input image was divided into 25 tiles (1024 × 1024 pixels) with a 100-pixel overlap.

**Table 5:** Performance comparison and computational cost of each processing step.

Dataset	Pattern	Method	Time [s]	Precision	Recall	IoU
OMU	(1)	2D-only	96.98	0.6090	0.7185	0.4917
	(2)	+Clustering	187.90	0.7469	0.7001	0.5659
	(3)	+Cylinder	290.79	0.6373	**0.9340**	0.6098
	(4)	+Background	848.03	0.8310	0.9230	0.7771
	(5)	Proposed	935.62	**0.9502**	0.9014	**0.8607**
JM	(1)	2D-only	77.05	0.6623	0.7278	0.5308
	(2)	+Clustering	106.62	0.7617	0.7117	0.5821
	(3)	+Cylinder	170.63	0.4671	**0.9283**	0.4508
	(4)	+Background	505.39	0.7546	0.8941	0.6927
	(5)	Proposed	571.58	**0.8912**	0.9028	**0.8132**

Note: The best value in each metric column (Precision/Recall/IoU) is shown in bold.

**Table 6:** Performance comparison among DUFOMap, BeautyMap, and the proposed method (Osaka Metropolitan University, Sugimoto Campus).

Method	Total Time [s]	Precision	Recall	IoU
DUFOMap	303.01	-	0.9316	-
BeautyMap	1562.37	-	0.7343	-
Ours (YOLOv8)	935.62	0.9502	0.9014	0.8607

**Table 7:** Performance comparison among DUFOMap, BeautyMap, and the proposed method (Jinaimachi, Tondabayashi City, Osaka Prefecture, Japan).

Method	Total Time [s]	Precision	Recall	IoU
DUFOMap	1842.68	-	0.6479	-
BeautyMap	4936.39	-	0.5226	-
Ours (YOLOv8)	571.58	0.8912	0.9028	0.8132

## Data Availability

The annotated human point cloud dataset (human noise labels only) is publicly available on GitHub: https://github.com/omu-geolab/AnnotationDatasetForHumanNoiseRemoval (accessed on 29 December 2025). The code used to implement the proposed method can be provided by the authors upon reasonable request.

## Abbreviations

The following abbreviations are used in this manuscript:

CNN	Convolutional Neural Network
COCO	Common Objects in Context
DBSCAN	Density-Based Spatial Clustering of Applications with Noise
FN	False Negative
FP	False Positive
FPS	Frames Per Second
GT	Ground Truth
IoU	Intersection over Union
KDE	Kernel Density Estimation
LiDAR	Light Detection and Ranging
Mask R-CNN	Mask Region-Based Convolutional Neural Network
MMS	Mobile Mapping System
PCA	Principal Component Analysis
RANSAC	Random Sample Consensus
RGB	Red-Green-Blue
RMSE	Root Mean Square Error
TLS	Terrestrial Laser Scanner
TP	True Positive
YOLO	You Only Look Once

## References

[1] Remondino F. (2011). Heritage Recording and 3D Modeling with Photogrammetry and 3D Scanning. Remote Sens..

[2] Yang S., Hou M., Li S. (2023). Three-Dimensional Point Cloud Semantic Segmentation for Cultural Heritage: A Comprehensive Review. Remote Sens..

[3] Zhang C., Zhang X., Lao M., Jiang T., Xu X., Li W., Zhang F., Chen L. (2025). Deep Learning for Point Cloud Denoising: A Survey. arXiv.

[4] Lin T.-Y., Maire M., Belongie S., Hays J., Perona P., Ramanan D., Dollár P., Zitnick C.L., Fleet D., Pajdla T., Schiele B., Tuytelaars T. (2014). Microsoft COCO: Common Objects in Context. Computer Vision—ECCV 2014.

[5] Schubert E., Sander J., Ester M., Kriegel H.P., Xu X. (2017). DBSCAN Revisited, Revisited: Why and How You Should (Still) Use DBSCAN. ACM Trans Database Syst.

[6] Guo Y., Wang H., Hu Q., Liu H., Liu L., Bennamoun M. (2021). Deep Learning for 3D Point Clouds: A Survey. IEEE Trans. Pattern Anal. Mach. Intell..

[7] Charles R.Q., Su H., Kaichun M., Guibas L.J. PointNet: Deep Learning on Point Sets for 3D Classification and Segmentation. Proceedings of the 2017 IEEE Conference on Computer Vision and Pattern Recognition (CVPR).

[8] Hu Q., Yang B., Xie L., Rosa S., Guo Y., Wang Z., Trigoni N., Markham A. RandLA-Net: Efficient Semantic Segmentation of Large-Scale Point Clouds. Proceedings of the 2020 IEEE/CVF Conference on Computer Vision and Pattern Recognition (CVPR).

[9] Thomas H., Qi C.R., Deschaud J.-E., Marcotegui B., Goulette F., Guibas L. KPConv: Flexible and Deformable Convolution for Point Clouds. Proceedings of the 2019 IEEE/CVF International Conference on Computer Vision (ICCV).

[10] Zhao H., Jiang L., Jia J., Torr P., Koltun V. Point Transformer. Proceedings of the 2021 IEEE/CVF International Conference on Computer Vision (ICCV).

[11] Lai X., Liu J., Jiang L., Wang L., Zhao H., Liu S., Qi X., Jia J. Stratified Transformer for 3D Point Cloud Segmentation. Proceedings of the 2022 IEEE/CVF Conference on Computer Vision and Pattern Recognition (CVPR).

[12] Choy C., Gwak J., Savarese S. 4D Spatio-Temporal ConvNets: Minkowski Convolutional Neural Networks. Proceedings of the 2019 IEEE/CVF Conference on Computer Vision and Pattern Recognition (CVPR).

[13] Qian G., Li Y., Peng H., Mai J., Al Kader Hammoud H.A., Elhoseiny M., Ghanem B. (2022). PointNeXt: Revisiting PointNet++ with Improved Training and Scaling Strategies. Proceedings of the 36th International Conference on Neural Information Processing Systems.

[14] Wu X., Jiang L., Wang P.-S., Liu Z., Liu X., Qiao Y., Ouyang W., He T., Zhao H. (2023). Point Transformer V3: Simpler, Faster, Stronger. arXiv.

[15] He K., Gkioxari G., Dollar P., Girshick R. Mask R-CNN. Proceedings of the 2017 IEEE International Conference on Computer Vision (ICCV).

[16] Redmon J., Divvala S., Girshick R., Farhadi A. You Only Look Once: Unified, Real-Time Object Detection. Proceedings of the 2016 IEEE Conference on Computer Vision and Pattern Recognition (CVPR).

[17] Ultralytics YOLOv8—Documentation. https://docs.ultralytics.com/models/yolov8/.

[18] Chen L.-C., Zhu Y., Papandreou G., Schroff F., Adam H., Ferrari V., Hebert M., Sminchisescu C., Weiss Y. (2018). Encoder-Decoder with Atrous Separable Convolution for Semantic Image Segmentation. Computer Vision—ECCV 2018.

[19] Carion N., Massa F., Synnaeve G., Usunier N., Kirillov A., Zagoruyko S., Vedaldi A., Bischof H., Brox T., Frahm J.-M. (2020). End-to-End Object Detection with Transformers. Computer Vision—ECCV 2020.

[20] Cheng B., Misra I., Schwing A.G., Kirillov A., Girdhar R. (2021). Masked-Attention Mask Transformer for Universal Image Segmentation. arXiv.

[21] Kirillov A., Mintun E., Ravi N., Mao H., Rolland C., Gustafson L., Xiao T., Whitehead S., Berg A.C., Lo W.-Y. (2023). Segment Anything. arXiv.

[22] Qi C.R., Liu W., Wu C., Su H., Guibas L.J. Frustum PointNets for 3D Object Detection from RGB-D Data. Proceedings of the 2018 IEEE/CVF Conference on Computer Vision and Pattern Recognition.

[23] Shin K., Kwon Y.P., Tomizuka M. RoarNet: A Robust 3D Object Detection Based on RegiOn Approximation Refinement. Proceedings of the 2019 IEEE Intelligent Vehicles Symposium (IV).

[24] Vora S., Lang A.H., Helou B., Beijbom O. PointPainting: Sequential Fusion for 3D Object Detection. Proceedings of the 2020 IEEE/CVF Conference on Computer Vision and Pattern Recognition (CVPR).

[25] Weinmann M., Jutzi B., Mallet C. (2017). Geometric Features and Their Relevance for 3D Point Cloud Classification. ISPRS Ann. Photogramm. Remote Sens. Spat. Inf. Sci..

[26] Sander J., Ester M., Kriegel H.-P., Xu X. (1998). Density-Based Clustering in Spatial Databases: The Algorithm GDBSCAN and Its Applications. Data Min. Knowl. Discov..

[27] Yang C.-K., Chen M.-H., Chuang Y.-Y., Lin Y.-Y. (2023). 2D-3D Interlaced Transformer for Point Cloud Segmentation with Scene-Level Supervision. arXiv.

[28] Peng S., Genova K., Jiang C.M., Tagliasacchi A., Pollefeys M., Funkhouser T. (2022). OpenScene: 3D Scene Understanding with Open Vocabularies. arXiv.

[29] Yue H., Wang Q., Zhang M., Xue Y., Lu L. (2025). 2D–3D Fusion Approach for Improved Point Cloud Segmentation. Autom. Constr..

[30] Habibiroudkenar P., Ojala R., Tammi K. (2024). DynaHull: Density-Centric Dynamic Point Filtering in Point Clouds. J. Intell. Robot. Syst..

[31] Duberg D., Zhang Q., Jia M., Jensfelt P. (2024). DUFOMap: Efficient Dynamic Awareness Mapping. IEEE Robot. Autom. Lett..

[32] Jia M., Zhang Q., Yang B., Wu J., Liu M., Jensfelt P. (2024). BeautyMap: Binary-Encoded Adaptable Ground Matrix for Dynamic Points Removal in Global Maps. IEEE Robot. Autom. Lett..

[33] Fischler M.A., Bolles R.C. (1981). Random Sample Consensus: A Paradigm for Model Fitting with Applications to Image Analysis and Automated Cartography. Commun ACM.

[34] Pauly M., Keiser R., Kobbelt L.P., Gross M. (2003). Shape Modeling with Point-Sampled Geometry. ACM Trans. Graph..

[35] Sun P., Kretzschmar H., Dotiwalla X., Chouard A., Patnaik V., Tsui P., Guo J., Zhou Y., Chai Y., Caine B. Scalability in Perception for Autonomous Driving: Waymo Open Dataset. Proceedings of the 2020 IEEE/CVF Conference on Computer Vision and Pattern Recognition (CVPR).

[36] Liu Y., Wang X., Hu E., Wang A., Shiri B., Lin W. (2025). VNDHR: Variational Single Nighttime Image Dehazing for Enhancing Visibility in Intelligent Transportation Systems via Hybrid Regularization. IEEE Trans. Intell. Transp. Syst..

[37] Li Q., Du Q., Tian L., Liao W., Lu G. (2025). Enhanced Semantic Segmentation of LiDAR Point Clouds Using Projection-Based Deep Learning Networks. IEEE Trans. Geosci. Remote Sens..

